# Area Deprivation and Clinical Biomarkers of Inflammation in Cancer Survivors of the National Institutes of Health *All of Us* Research Program

**DOI:** 10.1002/cam4.70784

**Published:** 2025-04-04

**Authors:** Cecily A. Byrne, Vanessa M. Oddo, Evgenia Karayeva, Greg Kopetsky, Sage Kim

**Affiliations:** ^1^ Cancer Health Equity and Career Development Program University of Illinois Chicago Chicago Illinois USA; ^2^ Kinesiology and Nutrition Department University of Illinois Chicago Chicago Illinois USA; ^3^ School of Public Health University of Illinois Chicago Chicago Illinois USA

**Keywords:** albumin, area deprivation index, cancer survivorship, c‐reactive protein, inflammation, neutrophil‐to‐lymphocyte ratio

## Abstract

**Background:**

High neighborhood deprivation is linked to increased cancer and overall mortality. Prior studies demonstrated higher inflammation in people from high deprivation areas. The area deprivation index (ADI) is a composite measure of income, education, employment, and housing, which quantifies neighborhood deprivation. We used the *All of Us* dataset to test whether inflammation, measured via c‐reactive protein (CRP), albumin, and the neutrophil‐to‐lymphocyte ratio (NLR), differs by ADI in cancer survivors.

**Methods:**

Our sample included individuals with a history of lung, breast, prostate, and colorectal cancer, filtered for the presence of the inflammatory biomarkers. We used quartiles of ADI based on 3‐digit zip code and biomarkers from electronic health records. We estimated the association between ADI and inflammation using adjusted logistic regression (*n* = 690 for CRP; *n* = 4242 for albumin; *n* = 5183 for NLR).

**Results:**

The sample had a mean age of 66.2 ± 10.1 years, 63.0% were female, and 86.8% were White. Mean CRP (11.5 ± 17.5 mg/L) and NLR (3.6 ± 2.2) indicated moderate to high inflammation. In the fully adjusted model, there were 2.04 (95% CI:1.02, 4.11) and 2.17 higher odds (95% CI:1.16, 4.13) of elevated CRP when comparing quartile 4 and quartile 3, respectively, to the lowest ADI quartile. Regression models were not significant for albumin or NLR.

**Conclusion:**

Area deprivation is associated with CRP, a marker of stress that may lead to a higher risk of chronic diseases among cancer survivors. Future studies using a sample of cancer survivors with a wider range of ADI may help to strengthen this association.

## 1 Introduction

Lung, breast, prostate, and colorectal cancers are the four most frequently diagnosed cancers in the United States and account for nearly 50% of all new cancer cases and close to 40% of cancer deaths [1]. Exposure to neighborhood disadvantage contributes to the stress response, which may affect cancer development, progression, and metastasis through stress‐related inflammation and a chronic inflammatory environment [2]. Racial/ethnic minorities are often disproportionately affected by neighborhood disadvantage, while having higher all‐cancer mortality rates compared to White individuals at all socioeconomic deprivation levels [3].

Neighborhood disadvantage is often measured using the area deprivation index (ADI) which includes neighborhood‐level income, education, employment, and housing conditions. ADI is associated with poorer health outcomes, including all‐cause and cancer‐specific mortality and risk for 30‐day hospital readmissions in individuals residing in more deprived areas [3, 4, 5, 6]. In nonmetastatic breast, prostate, lung, and colorectal cancer, individuals from the most deprived neighborhoods experienced worse overall and cancer‐specific survival compared to those from the least deprived neighborhoods [7]. Thus, a better understanding of the association between ADI and stress‐evoked inflammation among individuals with cancer is important for developing multilevel interventions.

ADI may affect cancer‐related health through several mechanisms, including exposure to social stress, leading to the activation of the hypothalamic–pituitary–adrenal (HPA) axis and immune dysregulation [2, 8, 9]. Stress‐induced dysregulation, often measured via c‐reactive protein (CRP), albumin, and neutrophil‐to‐lymphocyte ratio (NLR), may affect cancer initiation, progression, and metastasis through a chronic inflammatory environment [2]. Studies of individuals without cancer find that living in the most disadvantaged areas is associated with higher inflammation, measured by CRP [10, 11, 12, 13, 14]. In a study utilizing two cohorts of health professionals, higher neighborhood socioeconomic status (SES) was inversely associated with CRP and overall inflammation score [15].

Despite being a plausible mechanism, no prior studies have investigated ADI in relation to inflammation among cancer survivors. This study aimed to fill that gap in the literature by investigating the association between ADI and inflammatory biomarkers among lung, breast, prostate, and colorectal cancer survivors from the National Institutes of Health (NIH) *All of Us* Research Program. We hypothesized that individuals from high deprivation areas would have higher levels of inflammation, particularly CRP, compared to individuals from low deprivation areas. Secondarily, we also explored the association between ADI and two other stress biomarkers, NLR and albumin, in this sample of cancer survivors.

## 2 Methods

### 2.1 Overview

This cross‐sectional study is a secondary analysis of deidentified data available from the NIH *All of Us* Research Program, an initiative to recruit individuals underrepresented in biomedical research to understand how the environment, lifestyle, and biology influence health and disease across diverse populations [16]. Version 7 of the Curated Data Repository, Controlled Tier, currently includes over 413,000 participants, including 75% individuals underrepresented in biomedical research (e.g., <high school education, annual income < $25,000, sexual and gender minorities) and 45% racial/ethnic minorities [17]. In addition, over 287,000 individuals (69.4%) have provided access to their electronic health record (EHR) data. The *All of Us* Research Program is approved by the NIH Institutional Review Board. All participants complete informed consent upon enrollment and consent to the collection and use of their data.

### 2.2 Analytic Sample

**FIGURE 1:**
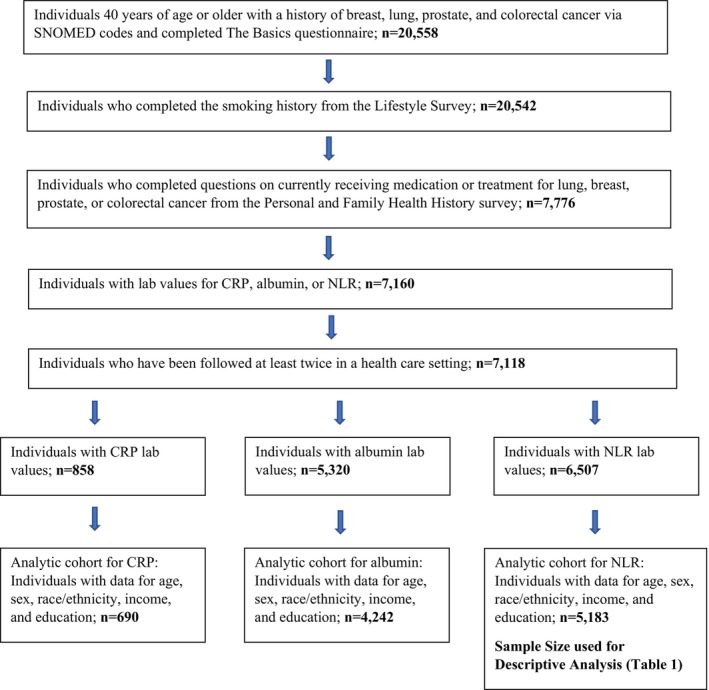
Flow chart of the derivation of analytic samples for individuals with CRP, albumin, and NLR. CRP, c‐reactive protein; NLR, neutrophil‐to‐lymphocyte ratio; SNOMED, Systematized Nomenclature of Medicine codes.

We used version 7 of the Controlled Tier data to extract our sample of lung, breast, prostate, and colorectal cancer survivors aged 40 years old or older (*n* = 20,558) using Systematized Nomenclature of Medicine (SNOMED) codes, which is standardized terminology used to link various diagnosis codes [18] from the EHR. Cancer survivors are defined by the American Cancer Society as any individual who has “ever been diagnosed with cancer no matter where they are in the course of their disease.” This may include individuals currently living with cancer or those in remission [19]. Participants were included if they completed the required basic demographic questions from the Basics Survey (*n* = 20,558), smoking history from the Lifestyle Survey (*n* = 20,542), questions on currently receiving medication or treatment for lung, breast, prostate, or colorectal cancer from the Personal and Family Health History survey (*n* = 7776), had lab values for CRP, albumin, or NLR (*n* = 7160), and were followed at least twice in a health care setting (*n* = 7118). Of 7118 individuals, 858 had CRP lab values; 5320 had albumin, and 6507 had NLR lab values. Individuals were further excluded if they were missing data on covariates (described below) for model 1 logistic regression for a final analytic sample of *n* = 690 for CRP, *n* = 4242 for albumin, and *n* = 5183 for NLR. See Figure 1 for a detailed flow chart on the derivation of the analytic samples for each of the inflammatory biomarkers.

### 2.3 Dependent Variables

In these analyses, inflammation was primarily measured via CRP, an acute‐phase protein, which increases in response to cytokine release and is an indicator of current inflammation and stress exposure [20]. CRP has a half‐life of 19 h [21]. Secondarily, inflammation was measured via albumin and NLR due to their clinical availability in the EHR and previous investigation as inflammatory biomarkers [22, 23]. Albumin is a negative acute‐phase protein that decreases with inflammation and malnutrition, among other factors, but is routinely available in the clinical setting [24]. Albumin has a long half‐life of approximately 21 days [25], which would reflect nutrition and inflammation status 3 weeks before [24]. NLR is a measurement of innate to adaptive immunity and pro to anti‐inflammatory response [26]. NLR is an indicator of neuroendocrine stress [27], and can be easily calculated from a routine complete blood count. While studies have found that NLR [28, 29] and albumin [24, 28, 29] are prognostic indicators of mortality in cancer, CRP may be a more sensitive indicator of social stress [20] experienced by individuals from deprived areas, and therefore, analyzed as the primary indicator of inflammation.

CRP, albumin, and NLR values within 12 months of enrollment or completion of the first core survey (i.e., The Basics) were extracted from EHR using standardized Logical Observation Identifiers Names and Codes (LOINC) vocabulary, which identifies lab measurement orders and results [30]. The values were converted to standardized units (e.g., for CRP, converted to mg/L). CRP values greater than 100 mg/L and NLR values greater than 23 were excluded as these values are indicative of acute bacterial infections, viral infections, systemic vasculitis, major trauma [31] and critical systemic inflammation, polytrauma, major surgery, or terminal cancer [27]. Albumin values greater than 6 were excluded as these values were clinical outliers upon graphical representation and may represent dehydration [32]. For each participant, if multiple values were available for a given biomarker, the mean was calculated and utilized. CRP, albumin, and NLR were analyzed as categorical variables based on previously established cutoffs with CRP greater than 3 mg/L [11, 33, 34], albumin less than 3.5 g/dL [24, 28, 29], and NLR greater than or equal to 3 [27, 28, 29, 35] indicating inflammation.

### 2.4 Independent Variables

We used ADI calculated at the 3‐digit zip code level as available from the *All of Us* controlled tier data. The *All of Us* ADI used in this study is comprised of six indicators from the U.S. Census, American Community Survey (ACS) 5‐year estimates for 2013–2017: median income, % of households receiving public assistance, % of the population with at least a high school education, % of the population below poverty, % vacant housing, and % of the population with no health insurance. It corresponds to the population‐weighted average of the index for the census tracts covered by the 3‐digit zip code tabulation, and scores range from 0 to 1, with higher scores indicating more deprivation [17]. To note, the *All of Us* ADI is computed differently than the Neighborhood Atlas ADI, which uses 17 measures at the census block‐group level [36, 37].

ADI was analyzed as both a continuous and categorical variable, with below the median indicating less deprivation and above the median indicating more deprivation. ADI quartiles were also analyzed, with Quartile 1 (Q1) which indicates the least deprivation (ADI < 0.2684, < 25th percentile) serving as the reference in regression models. Quartile 2 (Q2) consisted of ADI between 0.2684 and < 0.2943 (25th—50th percentile); Quartile 3 (Q3) ranges between 0.2943 and < 0.3352 (50th—75th percentile); and quartile 4 (Q4) indicates the most deprivation (ADI ≥ 0.3352, ≥ 75th percentile).

### 2.5 Covariates and Confounders

Demographic variables extracted from survey data included date of birth, sex, race/ethnicity, income, education level, insurance status, employment status, and smoking and alcohol history. Age was calculated by subtracting the date of birth from the first date of survey completion. Body mass index (BMI) was extracted from the EHR as a measurement using standardized LOINC vocabulary. BMI was collected within 12 months of survey completion, which was categorized into four groups: underweight, normal, overweight, and obesity [38]. Data on current treatment for lung, breast, prostate, or colorectal cancer was extracted from the Personal and Family Health History survey.

Data was collected on medications and comorbidities previously reported or controlled for in studies investigating CRP and NLR using the National Health and Nutrition Examination Survey (NHANES) and Health and Retirement Study (HRS) data [23, 39, 40, 41]. All topical medications, as well as those administered via the eyes or ears, were excluded. Medications known to affect inflammation levels were categorized as either an immunostimulant, immunosuppressant, steroid, antibiotic, or aspirin based on standardized LOINC codes. Only those medications prescribed within 12 months of survey completion were categorized and used in logistic regression to parallel lab values for CRP, albumin, and NLR. Diabetes, cardiovascular disease, and hypertension were categorized based on standardized SNOMED codes, and diagnosis codes were reviewed to ensure consistency. White blood cells (WBC) were extracted from EHR and categorized as low (< 4000 cells/μL), normal (4000–11,000 cells/μL), or high (> 11,000 cells/μL) as elevated levels are indicative of infections, inflammation, malignancies, or hereditary disorders [42]. WBC greater than 100,000 cells/μL were excluded due to hyperleukocytosis [42], and only those values within 12 months of survey completion were utilized.

### 2.6 Statistical Analysis

Outcome variables were analyzed for normality, and ANOVA (independent t‐tests) or Kruskal–Wallis rank sum tests (Mann–Whitney U test) were used to evaluate differences in continuous variables by ADI. Chi‐square tests were used to assess differences in proportional distributions of categorized CRP, albumin, and NLR by ADI quartile.

We used logistic regression to test the association between ADI and elevated CRP (> 3 mg/L), low albumin (< 3.5 g/dL), and elevated NLR (≥ 3) after controlling for demographics (Model 1); current cancer treatment status, comorbidities, medications, smoking status, and BMI (Model 2); and WBC (Model 3). Sensitivity analysis was conducted to test the association between ADI and CRP in individuals not currently receiving medications or treatment for cancer. All statistical analyses were conducted using the R statistical program within the Jupyter Notebook with *p* < 0.05 indicating statistical significance.

## 3 Results

The primary analytic sample included *n* = 690 for CRP, *n* = 4,242 for albumin, and *n* = 5,183 for NLR. Table 1 describes sample characteristics. The overall sample was 63.0% female and 86.8% White. The mean age was 66.2 ± 10.1 years. The majority of the sample had some college or completed college education (90.6%), had medical insurance (99.2%), were retired (54.6%), and 41.7% had incomes over $100,000. Most participants never smoked (54.6%) and were current alcohol drinkers (80.8%). The mean BMI was 28.8 ± 6.1 kg/m^2^. Nearly 40% of the sample were currently receiving medications or treatment for lung, breast, prostate, or colorectal cancer. The mean time since cancer diagnosis was 5.3 ± 5.6 years before enrollment in the study, with a wide range of time from 40 years before to 5 years after enrollment in the research program.

**TABLE 1:** Demographics and inflammatory biomarkers of analytic sample by area deprivation index, *n* = 5183^a^.

	Mean ± standard deviation or *N* (%)				
	Overall data	Area deprivation index			
		Q1 (Least deprived) ADI < 0.2684	Q2 ADI ≥ 0.2684 & < 0.2943	Q3 ADI ≥ 0.2943 & < 0.3352	Q4 (Most deprived) ADI ≥ 0.3352
Age (years)***	66.2 ± 10.1	66.8 ± 9.9	66.5 ± 9.7	66.1 ± 10.1	65.2 ± 10.5
Sex***					
Female	3264 (63.0)	784 (56.4)	901 (65.0)	803 (65.7)	776 (65.7)
Male	1917 (37.0)	606 (43.6)	485 (35.0)	420 (34.3)	406 (34.3)
Race/ethnicity***					
Black	307 (5.9)	24 (1.7)	63 (4.6)	62 (5.1)	158 (13.4)
White	4498 (86.8)	1297 (92.8)	1260 (91.2)	1059 (86.6)	882 (74.6)
Hispanic	186 (3.6)	< 20 (< 2.0)	> 22 (> 2.0)	60 (4.9)	84 (7.1)
Other	192 (3.7)	56 (4.0)	36 (2.6)	42 (3.4)	58 (4.9)
Education***					
≤ High school/GED	489 (9.4)	68 (4.9)	136 (9.8)	168 (13.7)	117 (9.9)
≥ Some college	4694 (90.6)	1323 (95.1)	1251 (90.2)	1055 (86.3)	1065 (90.1)
Income***					
<$25,000	536 (10.3)	65 (4.7)	142 (10.2)	172 (14.1)	157 (13.3)
$25,000–< 50,000	865 (16.7)	150 (10.8)	265 (19.1)	231 (18.9)	219 (18.5)
$50,000–$100,000	1621 (31.3)	413 (29.7)	446 (32.2)	399 (32.6)	363 (30.7)
>$100,000	2161 (41.7)	763 (54.8)	534 (38.5)	421 (34.4)	443 (37.5)
Employment***^b^					
Employed	1784 (34.6)	521 (37.8)	428 (31.2)	416 (34.2)	419 (35.8)
Not employed	553 (10.7)	117 (8.4)	163 (11.9)	116 (9.6)	157 (13.4)
Retired	2813 (54.6)	746 (53.8)	786 (56.9)	682 (56.2)	599 (50.8)
BMI category***					
Underweight/normal	1479 (28.5)	400 (28.8)	379 (27.3)	336 (27.5)	364 (30.8)
Overweight	1864 (36.0)	556 (39.9)	479 (34.5)	432 (35.3)	397 (33.6)
Obese	1840 (35.5)	435 (31.3)	529 (38.1)	455 (37.2)	421 (35.6)
Smoking status^c^					
Current	245 (4.8)	44 (3.2)	69 (5.1)	70 (5.8)	62 (5.4)
Former	2067 (40.6)	571 (41.7)	556 (40.9)	479 (39.7)	461 (39.8)
Never	2779 (54.6)	753 (55.1)	734 (54.0)	657 (54.5)	635 (54.8)
Alcohol status***^d^					
Current	4136 (80.8)	1158 (83.9)	1118 (81.5)	951 (79.1)	909 (77.9)
Former	822 (16.0)	197 (14.3)	216 (15.8)	207 (17.2)	202 (17.3)
Never	163 (3.2)	25 (1.8)	37 (2.7)	45 (3.7)	56 (4.8)
Currently receiving medications or treatment for cancer^e^					
Yes	2060 (39.9)				
No	3107 (60.1)				
Time since diagnosis (years)	5.3 ± 5.6 (−5.0–40.0)				
CRP (mg/L; *n* = 858)**^f^	11.5 ± 17.5 (0.04–95.7)	10.7 ± 19.0	11.2 ± 16.3	13.5 ± 18.6	10.2 ± 15.8
NLR (*n* = 6507)****^f^	3.6 ± 2.2 (0.1–22.5)	3.5 ± 2.1	3.7 ± 2.1	3.7 ± 2.3	3.4 ± 2.2
Albumin (g/dL; *n* = 5320)**	4.10 ± 0.40 (1.99–5.20)	4.14 ± 0.38	4.09 ± 0.39	4.03 ± 0.42	4.12 ± 0.40
WBC (*n* = 5425)****^f^ (thousand/μL)	6.8 ± 3.1 (0.002–61.2)	6.9 ± 3.0	6.8 ± 2.7	7.0 ± 2.9	6.4 ± 3.6

Abbreviations: BMI, body mass index; CRP, c‐reactive protein; GED, general education diploma; NLR, neutrophil‐to‐lymphocyte ratio; Q1, quartile 1; Q2, quartile 2; Q3, quartile 3; Q4, quartile 4; WBC, white blood cells.

^a^ Data presented for analytic sample (*n* = 5183) based on a logistic regression model for ADI and NLR, adjusting for age, sex, race/ethnicity, income, and education **p < 0.05; ***p < 0.001; ****p < 0.0001.

^b^ 33 missing.

^c^ 92 missing.

^d^ 62 missing.

^e^ 16 missing.

^f^ Analyzed by the Kruskal–Wallis rank sum test as not normally distributed.

Mean ADI of the sample (0.30 ± 0.06, range 0.16–0.49) and in the entire *All of Us* Research cohort (0.33 ± 0.06, range 0.15–0.63) indicated participants were from less deprived areas. Compared to participants from the least deprived areas (Q1), participants in the most deprived areas (Q4) consisted of a higher proportion of Black (13.4%) and Hispanic (7.6%) individuals, as well as individuals with less than a high school or high school education (13.7% for Q3 and 9.9% for Q4) and low income (14.1% for Q3 and 13.3% for Q4). Mean CRP (11.5 ± 17.5 mg/L) and NLR (3.6 ± 2.2) indicated moderate to high inflammation based on previously established cutoffs, whereas albumin was within normal limits (4.10 ± 0.40 g/dL).

Table 2 summarizes the inflammatory biomarkers stratified by current cancer treatment status. Albumin was significantly lower in those currently receiving medications or treatment for cancer (4.08 ± 0.41) compared to those who were not (4.12 ± 0.39; *p* < 0.001). NLR was significantly higher for those receiving medications or treatment for cancer (3.7 ± 2.3) compared to those who were not (3.4 ± 2.1; *p* < 0.0001). However, CRP was not significantly different between the two groups (*p* = 0.35).

**TABLE 2:** Inflammatory biomarkers by current cancer treatment status.

	Mean ± standard deviation	
	Currently receiving treatment/medication	Not currently receiving treatment/medication
CRP (mg/L; *n* = 828)^a^	12.4 (18.7)	10.5 (16.2)
Albumin (g/dL; *n* = 5151)**	4.08 (0.41)	4.12 (0.39)
NLR (*n* = 6299)^a^***	3.7 (2.3)	3.4 (2.1)

Abbreviations: CRP, c‐reactive protein; g/dL, grams per deciliter; mg/L, milligrams per liter; NLR, neutrophil to lymphocyte ratio.

^a^ Analyzed by Mann–Whitney U test as not normally distributed. **p < 0.001; ***p < 0.0001.

### 3.1 Regression Analyses

Table 3 shows three models describing the association between ADI quartile and high CRP (> 3 mg/L). In Model 1, after adjusting for basic demographics, Q3 was associated with 1.90 higher odds of elevated CRP (95% CI: 1.22, 2.98) with Q4 and Q2 trending in the same direction and nearly significant for inflammation. After adjusting for current cancer treatment status, BMI, smoking status, comorbidities, and medications (Model 2), the odds of inflammation were 2.13 (95% CI: 1.11, 4.13) and 2.18 (95% CI: 1.19, 4.01) higher in Q4 and Q3, respectively, compared to Q1. With further adjustment for WBC level to account for possible infection (Model 3), the odds of inflammation were attenuated but still significant for Q4 (OR: 2.04; 95% CI: 1.02, 4.11) and for Q3 (OR: 2.17; 95% CI: 1.16, 4.13) compared to Q1. The associations between ADI and albumin and NLR were not statistically significant in any of the adjusted models (Tables S1 and S2).

**TABLE 3:** Adjusted logistic regression for the association between area deprivation index and inflammation prevalence (CRP > 3 mg/L), *n* = 690^a^.

	Model 1^b^ (*n* = 690)	Model 2^c^ (*n* = 450)	Model 3^d^ (*n* = 430)
	OR (95% CI)	OR (95% CI)	OR (95% CI)
ADI Quartile 1 (ref)			
ADI Quartile 2	1.57 (0.99, 2.49)	1.32 (0.72, 2.43)	1.48 (0.79, 2.76)
ADI Quartile 3	1.90 (1.22, 2.98)	2.18 (1.19, 4.01)	2.17 (1.16, 4.13)
ADI Quartile 4 (most deprived)	1.42 (0.91, 2.23)	2.13 (1.11, 4.13)	2.04 (1.02, 4.11)

Abbreviations: ADI, area deprivation index; CI, confidence interval; OR, odds ratio.

^a^ *n* value based on model 1 for the association between area deprivation and CRP > 3 mg/L.

^b^ Controlling for sex, race/ethnicity, age, income, and education.

^c^ Controlling for model 1+ currently receiving anticancer treatment, smoking status, body mass index, medications, and comorbidities.

^d^ Controlling for model 2+ white blood cells.

Our results were robust to the sensitivity analyses testing the association between ADI and elevated CRP in only those individuals not currently receiving medication or treatment for cancer. In Models 1, 2, and 3, ADI Q4 (most deprivation) was associated with more than two times higher odds of inflammation (OR: 2.52; 95% CI: 1.04, 6.27 for Model 3; Table S3).

## 4 Discussion

To our knowledge, this is the first study to investigate ADI and inflammation in cancer survivors using the NIH *All of Us* Research Program. Lung, breast, prostate, and colorectal cancer survivors present with moderate to high levels of inflammation within 12 months of enrollment in the program, as indicated by mean CRP and NLR levels. In adjusted models, individuals from the most deprived areas (versus least) have higher CRP compared to individuals from the least deprived areas after controlling for demographics, current cancer treatment status, BMI, smoking history, comorbidities, medications, and WBC. This relationship between deprived areas and CRP remained in individuals not currently receiving medications or treatment for cancer.

While prior studies have not investigated this question among cancer survivors, our mean values for CRP were consistent with values for individuals with other chronic diseases, including diabetes [43] and cardiovascular disease [44], suggesting that individuals with a history of cancer, like those with chronic diseases, present with moderate to high levels of inflammation (based on previously established cutoffs). Notably, in our sample, nearly 40% of cancer survivors were still receiving medications or treatment for cancer, which may be contributing to inflammation, although CRP did not vary between the two groups.

Our adjusted results indicate that there is an association between living in higher ADI areas (Q3 and Q4) versus lower (Q1) and higher CRP levels among cancer survivors. While prior studies have not looked at ADI specifically, we can draw on studies that investigate disadvantage by neighborhood SES (e.g., income, education) based on census block group or concentrated disadvantage index, as well as “risky” neighborhoods based on crime or poverty [10, 11, 12, 13, 14, 15]. Consistent with our findings, in these studies, lower neighborhood SES or neighborhoods with high violence or poverty were associated with higher CRP levels in various populations (e.g., college students to midlife adults) [10, 11, 12, 13, 14, 15].

ADI may be associated with inflammation through various pathways, including social stress, health behaviors, and diet. The physical stressors, such as vacant buildings and neighborhood disinvestment, and social stressors, including violence and discrimination for individuals living in deprived areas [45, 46], which are often segregated and predominantly racially minoritized communities [47], may lead to biological stress responses resulting in increased inflammation [8, 48]. In addition, individuals living in deprived areas may engage in health behaviors that increase inflammation, such as smoking [49] and sedentary lifestyles due to the lack of safe and accessible places to exercise, which contribute to obesity and other metabolic conditions [11, 50]. Diet may also play a role; individuals living in more deprived areas may have a limited ability to access and afford healthy foods, including fresh fruits and vegetables [51] and instead rely on inexpensive, nutrient‐poor foods that affect the inflammatory response [52]. Finally, individuals from deprived areas may have limited access to healthcare [53, 54, 55] resulting in delayed care and may increase the risk for inflammation and the development of chronic disease [2, 56].

While the *All of Us* program is designed to oversample individuals underrepresented in biomedical research, our sample of cancer survivors was comprised of mostly White females who are highly educated and have high incomes and thus are compositionally different than the larger *All of Us* cohort (e.g., 45% racial/ethnic minorities, 75% underrepresented in biomedical research) [17]. In addition, individuals with a history of breast, lung, prostate, and colorectal cancer included in the *All of Us* (*n* = 20,558) were 68.8% White and 31.2% racial/ethnic minorities, which is not reflective of the overall racial composition in the entire cohort. Moreover, in the full *All of Us* cohort, ADI ranged from 0.15 to 0.65 compared to the range of 0.16–0.49 in our analytic sample, which was relatively low deprivation areas. This is due, in part, to the fact that lower proportions of racial/ethnic minority participants completed additional surveys (e.g., Personal and Family Health History survey) outside the core surveys (e.g., The Basics, Lifestyle), as well as consented to include data from their EHR and had at least two visits in a health care facility. A study investigating the completion of the Social Determinants of Health (SDOH) survey from the *All of Us* showed that 29.6% of participants provided any data via the SDOH survey. Of those who did respond to the survey, 74.6% were White compared to 44.8% White race in the nonrespondent group. Nearly 63% of respondents to the survey had college or graduate degrees compared to 36.0% with college or graduate degrees in the nonrespondent group, and 33.8% of respondents had high incomes (>$100,000) compared to 15.9% with high incomes in the nonrespondent group [57]. This mirrors the underrepresentation of minorities in many national surveys. Future investigation of this research question with a more diverse sample is warranted.

There are limitations to this study, including its cross‐sectional design; therefore, we cannot make causal inferences between ADI and inflammation. The sample investigated in this study consisted primarily of White, highly educated females with high incomes, so the results may not be generalizable to the overall population of cancer survivors. In addition, the *All of Us* ADI was based on a three‐digit zip code, which may not be specific to the neighborhoods or communities where individuals live, but rather an indicator of the county where the individual resides; therefore, the results may not be comparable to studies which use the ADI based on the census block group.

Inflammation was investigated within 12 months of enrollment in the program, but this period may not best capture longer‐term stress and inflammation. All data on demographics and current cancer treatment status are self‐reported via surveys and may be inaccurate and/or biased. The stage of cancer, which impacts inflammation levels, was not available from the EHR as this is not coded for reimbursement, and very few biopsy results (*n* = 48) are available to confirm the stage of cancer. In future studies, the stage of cancer should be controlled for as inflammation increases in late‐stage cancer [28, 58]. Nevertheless, key strengths of this study included the ability to analyze clinically available biomarkers of inflammation from EHR across the United States in relation to ADI and utilize more than one measurement for each biomarker when available.

## 5 Conclusion

These results suggest that individuals with a history of lung, breast, prostate, and colorectal cancer have higher inflammation, measured via CRP and NLR, within 12 months of enrollment in the NIH *All of Us* Research Program. Moreover, our study begins to suggest that area deprivation contributes to a biophysical stress response in cancer survivors, as evidenced by increased CRP in individuals from the most deprived areas, despite the current receipt of cancer treatment or not. Future studies using more diverse samples with cancer may help to strengthen the evidence supporting the association between ADI and inflammation.

## Author Contributions

**Cecily A. Byrne:** conceptualization (equal), data curation (equal), formal analysis (equal), methodology (equal), writing – original draft (equal), writing – review and editing (equal). **Vanessa M. Oddo:** conceptualization (equal), methodology (equal), supervision (equal), writing – review and editing (equal). **Evgenia Karayeva:** data curation (equal), formal analysis (equal), writing – review and editing (equal). **Greg Kopetsky:** data curation (equal), formal analysis (equal), writing – review and editing (equal). **Sage Kim:** conceptualization (equal), methodology (equal), supervision (equal), writing – review and editing (equal).

## Disclosure

Institutional Review Board Statement: The *All of Us* Research Program is approved by the NIH Institutional Review Board. The secondary analysis of publicly available, deidentified data that does not involve direct contact with participants does not constitute human subjects research as defined by The Common Rule (HHS: 45 CFR 46); therefore, further institutional review board approval is not warranted.

## Consent

All participants in the NIH *All of Us* Research Program complete informed consent upon enrollment and consent to the collection and use of their data.

## Conflicts of Interest

The authors declare no conflicts of interest.

## Supporting information


Appendix S1.

## Data Availability

This study used data from the *All of Us* Research Program's Controlled Tier Dataset 7, available to authorized users on the Researcher Workbench.
