# Supplementary material for: Area Deprivation and Clinical Biomarkers of Inflammation in Cancer Survivors of the National Institutes of Health All of Us Research Program
Source: Cancer Med. 2025 Apr 4;14(7):e70784. doi: 10.1002/cam4.70784 (PMC11971236; doi:10.1002/cam4.70784)
Supplement: Supplementary file 1 — Appendix S1. [file CAM4-14-e70784-s001.docx]

**Appendices**

**Supplementary Table 1: Adjusted Logistic Regression for the Association between Area Deprivation Index and Inflammation Prevalence (albumin <3.5 g/dL), n=4242**^†^

|  | **Model 1**^‡^  n=4242 | **Model 2**^§^  n=2145 | **Model 3**^¶^  n=2000 |
| --- | --- | --- | --- |
|  | **OR (95% CI)** | **OR (95% CI)** | **OR (95% CI)** |
| ADI Quartile 1 (ref) |  |  |  |
| ADI Quartile 2 | 1.20 (0.84, 1.71) | 1.02 (0.67, 1.57) | 1.04 (0.67, 1.62) |
| ADI Quartile 3 | 1.50 (1.06, 2.13) | 1.29 (0.85, 1.97) | 1.23 (0.80, 1.90) |
| ADI Quartile 4 (most deprived) | 1.10 (0.76, 1.59) | 1.16 (0.72, 1.84) | 1.11 (0.68, 1.81) |

ADI = area deprivation index; CI = confidence interval; OR = odds ratio

^†^ n value based on model 1 for the association between area deprivation and albumin <3.5 g/dL

^‡^ controlling for sex, race/ethnicity, age, income, and education

^§^ controlling for model 1 + currently receiving anti-cancer treatment, smoking status, body mass index, medications, and comorbidities

^¶^ controlling for model 2 + white blood cells

**Supplementary Table 2: Adjusted Logistic Regression for the Association between Area Deprivation Index and Inflammation Prevalence (NLR ≥ 3), n=5183**^†^

|  | **Model 1**^‡^  n=5183 | **Model 2**^§^  n=2426 | **Model 3**^¶^  n=2140 |
| --- | --- | --- | --- |
|  | **OR (95% CI)** | **OR (95% CI)** | **OR (95% CI)** |
| ADI Quartile 1 (ref) |  |  |  |
| ADI Quartile 2 | 1.19 (1.02, 1.39) | 1.15 (0.91, 1.44) | 1.12 (0.88, 1.43) |
| ADI Quartile 3 | 1.16 (0.99, 1.36) | 1.13 (0.89, 1.42) | 1.01 (0.79, 1.30) |
| ADI Quartile 4 (most deprived) | 0.96 (0.81, 1.13) | 1.15 (0.89, 1.49) | 1.06 (0.80, 1.40) |

ADI = area deprivation index; CI = confidence interval; OR = odds ratio

^†^ n value based on model 1 for the association between area deprivation and NLR **≥**3

^‡^ controlling for sex, race/ethnicity, age, income, and education

^§^ controlling for model 1 + currently receiving anti-cancer treatment, smoking status, body mass index, medications, and comorbidities

^¶^ controlling for model 2 + white blood cells

**Supplementary Table 3: Adjusted Logistic Regression for the Association between Area Deprivation Index and Inflammation Prevalence (CRP >3 mg/L) in Individuals Not Currently Receiving Cancer Treatments (n=264)**

|  | **Model 1***  n=264 | **Model 2****  n=263 | **Model 3*****  n=263 |
| --- | --- | --- | --- |
|  | **OR (95% CI)** | **OR (95% CI)** | **OR (95% CI)** |
| ADI Quartile 1 (ref) |  |  |  |
| ADI Quartile 2 | 1.54 (0.74, 3.26) | 1.65 (0.73, 3.76) | 1.78 (0.77, 4.13) |
| ADI Quartile 3 | 1.59 (0.79, 3.22) | 2.10 (0.95, 4.72) | 2.04 (0.91, 4.67) |
| ADI Quartile 4 (most deprived) | 2.42 (1.09, 5.53) | 2.61 (1.10, 6.34) | 2.52 (1.04, 6.27) |

ADI = area deprivation index; CI = confidence interval; OR = odds ratio

*controlling for sex, race/ethnicity, age, income, and education

**controlling for model 1 + smoking status, body mass index, medications, and comorbidities

*** controlling for model 2 + white blood cells
